# Patient Education Deficits and Medication Knowledge Gaps Among Post-Percutaneous Coronary Intervention Patients: A Cross-Sectional Study of Communication Quality and Adherence in Saudi Cardiac Care

**DOI:** 10.3390/healthcare14070891

**Published:** 2026-03-31

**Authors:** Muteb Aljuhani, Asrar S. Almutairi, Rayhanah R. Almutairi, Abdulaziz M. Alodhailah

**Affiliations:** 1 Department of Community Health, Mental and Psychiatric Nursing, Imam Mohammad Ibn Saud Islamic University (IMSIU), Riyadh 11564, Saudi Arabia; 2 Community and Psychiatric Mental Health Nursing Department, College of Nursing, Princess Nourah Bint Abdulrahman University, Riyadh 11671, Saudi Arabia; 3 Department of Medical-Surgical Nursing, College of Nursing, King Saud University, Riyadh 11451, Saudi Arabia

**Keywords:** patient education, medication knowledge, communication quality, percutaneous coronary intervention, medication adherence, nursing care, Saudi Arabia

## Abstract

**Background**: Effective patient education is fundamental to promoting medication adherence following percutaneous coronary intervention (PCI). However, the extent and nature of educational gaps in Middle Eastern cardiac care settings remain inadequately explored. This study examined medication-related communication quality, patient knowledge gaps, and their associations with antiplatelet adherence among Saudi adults post-PCI. **Methods**: A cross-sectional survey was conducted among 236 Saudi adults who had undergone PCI within the preceding 12 months at two tertiary cardiac centres in Riyadh, Saudi Arabia. Data were collected using a structured questionnaire assessing clarity of medication information (side effects, dosing, purpose), medication knowledge, and adherence measured via the Arabic Morisky Medication Adherence Scale-8 (MMAS-8). Descriptive statistics, bivariate analyses, and multivariable logistic regression examined associations between communication quality indicators, sociodemographic factors, and adherence outcomes. **Results**: Substantial educational deficits emerged: (1) 26.3% never received side-effect information, (2) 11.0% never learned medication purpose, and (3) 6.8% did not identify their antiplatelet medication type. Only 40.5% consistently received clear medication explanations during hospitalization. Understanding of medication instructions was inadequate, with 14.0% reporting that they never understood their instructions. Adherence was suboptimal, with 54.9% demonstrating low adherence (MMAS-8 score < 6). In adjusted logistic regression, never receiving side-effect information was associated with higher odds of low adherence (adjusted OR 2.14, 95% CI 1.18–3.89) after controlling for age, sex, education, time since PCI, diabetes, and home support. **Conclusions**: Significant gaps in medication education and patient–provider communication exist within Saudi cardiac care settings. These deficits represent potentially modifiable targets for nursing intervention. Structured, nurse-led educational protocols addressing medication purpose, side effects, and administration could be evaluated as strategies to enhance patient understanding and support antiplatelet adherence.

## 1. Introduction

Effective communication between healthcare providers and patients constitutes a cornerstone of quality care, particularly in the management of complex cardiovascular conditions requiring long-term pharmacotherapy [1]. Following percutaneous coronary intervention (PCI), patients are typically prescribed dual antiplatelet therapy (DAPT), commonly aspirin combined with a P2Y12 inhibitor (e.g., clopidogrel, ticagrelor, or prasugrel), to prevent stent thrombosis and recurrent cardiovascular events [2,3]. The clinical benefits of these medications, however, are contingent upon patients’ understanding of their prescribed regimens and their ability to adhere consistently over extended periods [4].

Patient education encompasses multiple dimensions, including information provision about medication purpose, dosing schedules, potential adverse effects, and strategies for managing missed doses [5]. Research consistently demonstrates that patients who receive comprehensive, clear medication counselling exhibit higher adherence rates and better clinical outcomes than those with limited understanding of their treatment regimens [6,7]. Conversely, inadequate communication has been linked to medication errors, unintentional non-adherence, and preventable hospitalizations [8].

Among patients with cardiovascular disease, knowledge deficits pose particular risks. Patients who do not understand why antiplatelet medications are prescribed, or who lack awareness of potential side effects, may discontinue therapy prematurely or fail to recognize warning signs requiring medical attention [9]. Studies from both Western and Middle Eastern healthcare systems have documented associations between medication knowledge, health literacy, and adherence behaviours. Western research and regional Saudi studies consistently demonstrate these associations [10,11,12]. However, evidence from Middle Eastern contexts remains limited, despite recognition that cultural, linguistic, and health system factors may uniquely shape patient–provider communication patterns in this region [12].

Saudi Arabia presents a distinctive context for examining medication education quality. The healthcare system has undergone rapid expansion, with substantial investments in tertiary cardiac centres and interventional cardiology services [13]. Health literacy among Saudi adults is relatively low, with nearly half scoring below average on standardized assessments, particularly among older and less-educated populations. Cultural factors, including family involvement in healthcare decisions and beliefs regarding divine predestination, may further influence how patients engage with medication information [14,15]. These considerations underscore the importance of empirically examining communication quality and knowledge gaps within Saudi cardiac care settings.

Nurses occupy a pivotal position in patient education, serving as the primary point of contact for discharge counselling and follow-up reinforcement [16]. Systematic reviews demonstrate that nurse-led educational interventions improve medication adherence across multiple chronic conditions, including cardiovascular disease [16]. However, to design effective interventions, it is first necessary to characterize the nature and extent of existing educational deficits. Pinpointing specific gaps, whether in side-effect information, dosing instructions, or basic medication knowledge, can guide the development of targeted, culturally appropriate nursing protocols.

Key concepts are defined as follows:

- Communication quality: Information provided by healthcare providers about medications (purpose, dosing, side effects).
- Medication knowledge: Patients’ understanding and recall of their medications.
- Health literacy: The ability to access, understand, and apply health information across contexts.
- Medication adherence: The extent to which patients follow prescribed medication regimens.

Most prior studies on post-PCI medication education have been conducted in North American and European cardiac centers, where health literacy is generally higher and healthcare systems differ from those in the Middle East. This is the first quantitative study to examine medication communication quality and its relationship to adherence among post-PCI patients in Saudi Arabia, a setting with distinct cultural factors (e.g., family involvement in decision-making, health literacy disparities) and healthcare characteristics (e.g., tertiary center throughput, emergency PCI prevalence). It addresses a critical evidence gap in the Gulf Cooperation Council region.

This study aimed to: (1) describe medication-related communication quality and patient knowledge among post-PCI patients in Saudi Arabia; (2) assess self-reported antiplatelet adherence; and (3) examine independent associations between communication quality indicators and low adherence after adjusting for potential confounders.

## 2. Materials and Methods

This study utilized a cross-sectional quantitative survey design to examine medication education quality and patient knowledge among post-PCI patients in Saudi Arabia. The cross-sectional approach enabled assessment of communication patterns and their associations with adherence at a single time point, providing foundational data to inform future interventional research [17]. The study is reported in accordance with the Strengthening the Reporting of Observational Studies in Epidemiology (STROBE) guidelines for cross-sectional studies (Supplementary Table S1).

Data were collected from two tertiary referral hospitals in Riyadh, Saudi Arabia. Both facilities serve as major cardiac centres offering comprehensive interventional cardiology services to diverse urban and semi-urban populations.

- Inclusion: Saudi adults aged ≥18 years who had undergone PCI within the preceding 12 months and were prescribed antiplatelet therapy (aspirin, clopidogrel, ticagrelor, or prasugrel, alone or in combination).
- Exclusion: Patients with documented cognitive impairment (as noted in medical records), severe psychiatric illness precluding informed consent, inability to provide informed consent in either Arabic or English (interpreters were not available).

The study was designed with a 95% confidence level (α = 0.05, two-sided) and 7% precision margin to estimate a population proportion. Based on regional literature indicating low adherence prevalence of approximately 50% in post-PCI populations, the formula: *n* = Z^2^ p(1 − p)/e^2^ was applied, where Z = 1.96 (critical value for 95% confidence), p = 0.50 (anticipated proportion), and e = 0.07 (precision margin), yielding a minimum sample of 196 participants. Accounting for an anticipated 15% incomplete response rate, we targeted recruitment of 230 patients. Our achieved *n* = 236 exceeded the target, providing adequate statistical power.

Sampling and recruitment: A consecutive sampling strategy was employed to recruit participants during routine cardiology follow-up visits. Trained research assistants (two registered nurses independent of the clinical care team) screened outpatient appointment lists daily and approached eligible patients in the waiting area. Of 302 patients approached, 47 declined participation (reasons: time constraints [*n* = 28], not interested [*n* = 15], feeling unwell [*n* = 4]) and 19 returned incomplete questionnaires (>20% missing items), yielding a final analytical sample of 236 (response rate: 78.1%). A comparison of demographic characteristics between responders and non-responders was not possible because detailed data on decliners were not collected; this is acknowledged as a limitation.

Data were collected using a structured questionnaire comprising the following components (full questionnaire provided in Supplementary Table S2):

1. Demographic and Clinical Profile: Items assessed age, sex, marital status, educational attainment, employment status, monthly household income, comorbidities (diabetes mellitus, hypertension, hyperlipidaemia), time since heart disease diagnosis, time since most recent PCI (categorized as ≤3 months, 4–6 months, 7–12 months), PCI indication (elective vs. acute coronary syndrome [ACS]/emergency), and current antiplatelet regimen (DAPT vs. single antiplatelet therapy).
2. Communication Quality Assessment: Six investigator-developed items were created based on a literature review of medication communication domains and expert consultation with three cardiologists and two cardiac nurses. Content validity review: Items underwent formal content validity review by a panel of 5 experts (cardiologists, *n* = 3; cardiac nurses, *n* = 2; health educator, *n* = 1) who rated relevance (1–4 scale) for measuring medication communication quality; all items achieved CVI (content validity index) ≥ 0.80, indicating excellent relevance. Translation and piloting: The Arabic version was produced via forward–backward translation by two independent bilingual researchers, reconciled by consensus, and pilot-tested with 20 post-PCI patients (not included in main sample) for clarity and comprehension; minor wording adjustments were made based on pilot feedback. Reliability: Internal consistency: Cronbach’s α = 0.72. Analytical rationale: Items were analyzed both as Likert-ordered responses (Never/Sometimes/Usually/Always) and dichotomized (Never vs. any information) to isolate patients receiving zero education versus any education, as this distinction has the highest clinical relevance for identifying education gaps. Ordinal analyses examining all four frequency levels are presented in Supplementary Materials (Supplementary Table S3): “Thinking about your care since your heart procedure, how often did healthcare providers…” followed by:
  (a) “…give you clear explanations about your medications during your hospital stay?”
  (b) “…tell you the purpose of any new medications?”
  (c) “…explain possible side effects of your medications?”
  (d) “…make sure you understood your medication instructions before leaving the hospital?”
  (e) “…ask whether you have someone at home to help you take your medications?”
  (f) “…ensure you see the same cardiac specialist at your follow-up visits?”
3. Medication Knowledge: Patients were asked: “What is the name of the antiplatelet (blood-thinning) medication you are currently taking to protect your heart stent?” Responses were categorized as: correct identification (named at least one prescribed antiplatelet correctly, verified against medical records), incorrect identification, or “don’t know.”
4. Morisky Medication Adherence Scale-8 (MMAS-8): This validated 8-item instrument measured self-reported adherence to antiplatelet therapy [18]. We used the Arabic MMAS-8 version validated in Saudi cardiovascular populations (permission obtained from MMAS Research LLC, license agreement dated 15 November 2022). The MMAS-8 is based on self-report, which may overestimate adherence due to social desirability bias; however, it remains the most widely used instrument in cardiac populations, enabling international comparison. Scoring classified participants as: high adherence (score = 8), medium adherence (score 6 to <8), or low adherence (score < 6). Internal consistency in this sample was Cronbach’s α = 0.68.

For primary analyses, adherence was dichotomized as low (<6) vs. medium/high (≥6) because the clinical distinction between non-adherence (score < 6, indicating problematic adherence) and adequate adherence (score ≥ 6) is most meaningful for practice. Sensitivity analyses examining ordinal relationships (three-level adherence: low, medium, high) yielded consistent findings and are presented in Supplementary Table S3. Participants missing ≥2 MMAS-8 items (*n* = 3) were excluded from adherence analyses.

Trained research assistants (independent of clinical staff) approached eligible patients during scheduled follow-up visits, explained study objectives, and obtained written informed consent. Questionnaires were administered in a private room adjacent to the clinic, with options for self-completion or interviewer-assisted completion for patients with limited literacy (*n* = 42, 17.8%). Survey completion required approximately 10–15 min. Clinical data (PCI indication, antiplatelet regimen) were extracted from electronic medical records with patient consent.

Descriptive statistics summarized categorical variables as frequencies and percentages; continuous variables (age, time since PCI) were summarized as means with standard deviations (SD) or medians with interquartile ranges (IQR) as appropriate. Missing data were reported per variable; no imputation was performed given low missingness (<3% per variable).

Bivariate analyses: Communication quality indicators were examined across adherence categories (low vs. medium/high combined) using chi-square tests or Fisher’s exact test where cell counts were <5. Effect sizes (Cramér’s V) were reported.

Multivariable analysis: A binary logistic regression model examined independent associations between communication quality indicators and low adherence (MMAS-8 < 6 vs. ≥6). Covariates were prespecified based on the literature and included: age (continuous), sex, educational attainment (no formal schooling, general education, higher education), time since PCI (≤3, 4–6, 7–12 months), diabetes mellitus, hypertension, home support availability, and continuity of care (same specialist). Adjusted odds ratios (aOR) with 95% confidence intervals (CI) were reported. Model fit was assessed using the Hosmer–Lemeshow test.

Model assumptions: Binary logistic regression assumptions were assessed as follows: (1) linearity of the logit for continuous predictors (age) was evaluated using Box-Tidwell plots; (2) independence of observations was confirmed by study design (each patient = one observation); (3) multicollinearity was assessed using variance inflation factors (VIF; all VIF < 2, indicating acceptable separation of predictors).

Sensitivity analyses stratified by time since PCI and PCI indication were conducted.

All analyses were performed using SPSS version 28 (IBM Corp., Armonk, NY, USA). Statistical significance was set at two-sided *p* < 0.05.

The study was conducted in accordance with the Declaration of Helsinki. Ethical approval was obtained from the Institutional Review Board of King Saud University (IRB No. 17/0174/IRB) and King Fahad Medical City (IRB No. 17-019E). All participants provided written informed consent prior to data collection. Participation was voluntary, and patients were assured that declining would not affect their clinical care. No identifying information was recorded on questionnaires; data were stored on password-protected servers accessible only to the research team.

## 3. Results

### 3.1. Participant Flow

**Figure 1:**
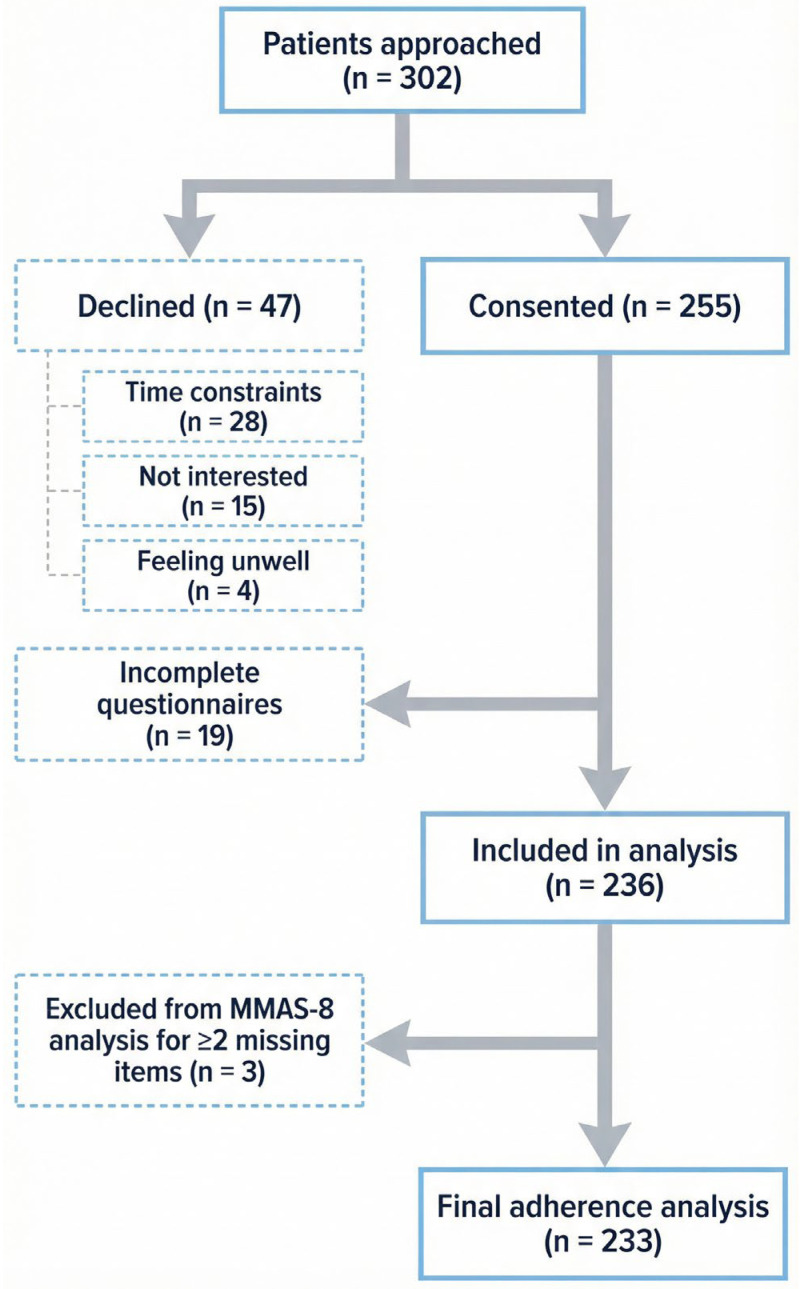
Participant Flow Diagram.

Of 302 patients screened, 236 were included in the final analysis (Figure 1). The most common reason for non-participation was time constraints (*n* = 28).

### 3.2. Sample Characteristics

The sample (*n* = 236) characteristics are presented in Table 1. Participants were predominantly male (73.7%), married (80.9%), and with a mean age of 58.4 years (SD 11.2). Educational attainment was generally low: 33.1% reported no formal schooling and 45.3% had completed general education only. The majority (69.5%) had pre-existing heart disease prior to the index PCI. Hypertension (51.3%) and diabetes mellitus (45.0%) were highly prevalent comorbidities.

### 3.3. Medication-Related Communication Quality

Substantial deficits in medication education were identified across multiple domains (Table 2)

Regarding clarity of medication explanations during hospitalization, only 40.3% of participants reported consistently (“Always”) receiving clear information, while 6.8% indicated they never received clear explanations. Information about medication purpose was similarly limited, with 39.4% always informed and 11.0% never informed.

The most pronounced deficit concerned side-effect communication. As shown in Table 2, more than one-quarter (26.3%, *n* = 62) of participants reported they had never been informed about potential adverse effects of their medications, while only 26.3% (*n* = 62) consistently received this information. Overall, 73.8% reported receiving side-effect information less than “always,” suggesting inconsistent counselling.

Understanding of medication instructions was inadequate for a substantial proportion of patients. While 27.1% reported always understanding their instructions, 14.0% acknowledged they never understood what they were told, and 39.8% understood only sometimes.

### 3.4. Medication Knowledge

Analysis of medication knowledge revealed that 16 participants (6.8%) did not know which type of antiplatelet medication they were prescribed. An additional 8 participants (3.4%) incorrectly identified their medication. This combined 10.2% with absent or incorrect knowledge indicates a notable gap in understanding of the medications central to their cardiovascular secondary prevention.

### 3.5. Continuity of Care

Consistent follow-up with the same cardiac specialist was reported by only 39.0% of participants as “always” occurring. Nearly one-fifth (18.6%) indicated they never saw the same specialist, and 22.9% only sometimes did.

### 3.6. Social Support for Medication Management

Home support for medication management was available consistently (“always”) to 48.3% of participants. However, 30.1% reported never having someone at home to assist with their medications.

### 3.7. Medication Adherence Outcomes

Adherence levels were concerning (Table 3). More than half of participants (54.9%) demonstrated low adherence on the MMAS-8, 35.2% showed medium adherence, and only 9.9. % achieved high adherence.

### 3.8. Bivariate Associations Between Communication Quality and Adherence

Table 4 presents bivariate associations between communication quality indicators and low adherence. Patients never informed about side effects had notably higher prevalence of low adherence (71.0%) compared to those who sometimes/usually/always received this information (49.1%, χ^2^ = 8.42, *p* = 0.004, Cramér’s V = 0.19). Similarly, those who never understood medication instructions showed higher low adherence rates (75.0% vs. 51.7%, χ^2^ = 6.18, *p* = 0.013, Cramér’s V = 0.16). Absence of home support was associated with low adherence (67.1% vs. 49.7%, χ^2^ = 4.92, *p* = 0.027, Cramér’s V = 0.15). Continuity of care showed a non-significant trend (χ^2^ = 3.21, *p* = 0.073, Cramér’s V = 0.12). Effect sizes (Cramér’s V) ranged from 0.12 to 0.19, indicating small to small-medium associations.

### 3.9. Multivariable Logistic Regression

In adjusted analysis, never receiving side-effect information was significantly associated with higher odds of low adherence (aOR 2.14, 95% CI 1.18–3.89, *p* = 0.012). Absence of home support was also independently associated with low adherence (aOR 1.72, 95% CI 1.01–2.94, *p* = 0.046). Never understanding medication instructions showed a trend toward association with low adherence but did not reach statistical significance (aOR 1.89, 95% CI 0.91–3.93, *p* = 0.087).

The multivariable model explained approximately 14% of variance in adherence (Nagelkerke R^2^ = 0.14), indicating that while communication quality and demographic factors are independent contributors to adherence, substantial additional variance is attributable to factors not captured in this cross-sectional survey, including patient beliefs about medication necessity and harm, perceived medication side effects, financial barriers (medication costs), healthcare system factors (appointment frequency, provider consistency), and individual psychosocial factors (depression, cognitive function). Table 5 presents adjusted associations between communication quality indicators and low adherence.

### 3.10. Sensitivity Analyses

Stratified analyses by time since PCI showed consistent patterns: the association between never receiving side-effect information and low adherence was observed across all time strata (aOR range 2.07–2.39), though confidence intervals widened in subgroups due to smaller sample sizes. Stratification by PCI indication (elective vs. ACS) showed similar patterns, with point estimates suggesting stronger associations in the ACS/emergency subgroup (aOR 2.48, *p* = 0.017) compared to elective PCI (aOR 1.89, *p* = 0.197), suggesting that education deficits may particularly impact adherence in patients with acute presentations (Supplementary Table S4).

## 4. Discussion

This study provides empirical data on medication education quality and patient knowledge gaps among Saudi adults following PCI. The findings reveal substantial deficits in multiple communication domains, with particularly notable gaps in side-effect education and medication understanding. After adjustment for potential confounders, never receiving information about medication side effects was independently associated with low adherence; a finding with potential implications for nursing practice and the design of patient education interventions.

### 4.1. Key Findings in Context

The finding that 26.3% of patients never received information about medication side effects indicates a substantial gap in medication education, specifically in patient-reported counselling about adverse effects [19]. Awareness of potential adverse effects is essential for enabling patients to recognize symptoms requiring medical attention and make informed decisions about treatment continuation. This gap is consistent with barriers to adherence reported elsewhere [20,21], though the cross-sectional design precludes determining whether communication deficits precede non-adherence in this cohort. Both poor education and low adherence may be influenced by unmeasured factors such as provider knowledge, workload constraints, patient health literacy, or health beliefs.

The observation that 6.8% of participants did not know their antiplatelet medication type (and an additional 3.4% incorrectly identified it) highlights a basic knowledge deficit. Without this fundamental knowledge, patients may have difficulty communicating accurately with healthcare providers, identifying potential drug interactions, or seeking appropriate care in emergency situations.

Comparisons with international literature highlight the significance of these findings [22]. Studies in Western populations generally report higher levels of medication knowledge and more consistent patient education practices [23,24]. The gaps identified in this Saudi cohort may reflect multiple factors, including healthcare system capacity constraints, cultural communication patterns, language considerations, and the rapid throughput typical of busy tertiary cardiac centres. Emergency PCI procedures, which constituted 60% of our sample, may particularly limit opportunities for comprehensive pre-procedure education.

The finding that only 39% of patients consistently saw the same cardiac specialist across follow-up visits suggests fragmentation in continuity of care. Discontinuity may impede the reinforcement of medication education over time and reduce opportunities for identifying and addressing adherence barriers [25]. This finding has implications for healthcare system organization and the potential value of nurse-led follow-up programs that ensure consistent patient–provider relationships.

Social support emerged as another important consideration [26]. While 48% of participants had consistent home support for medication management, nearly one-third (30%) had no such assistance. In Saudi Arabia, where family caregivers traditionally play significant roles in patient care, the absence of home support may particularly disadvantage patients in managing complex medication regimens. Our finding that absent home support was independently associated with low adherence supports the inclusion of caregivers in educational interventions where feasible.

### 4.2. Strengths and Limitations

Strengths of this study include the focus on a regionally important context with limited prior research on post-PCI medication counselling, a response rate of 78% among eligible participants from two major tertiary cardiac centers, use of a validated adherence instrument (MMAS-8), and comprehensive adjustment for plausible confounders (age, sex, education, comorbidities, time since PCI, home support, continuity of care) in multivariable analysis.

Several important limitations warrant acknowledgment. First, the cross-sectional design precludes causal inference; we cannot determine whether communication deficits preceded low adherence or whether both are influenced by unmeasured confounders. Prospective studies are needed to establish temporality.

Second, selection bias is likely. Recruitment at follow-up clinics excluded patients lost to follow-up who may have substantially lower adherence, potentially inflating adherence estimates relative to the broader post-PCI population. The sample was drawn from two tertiary cardiac centers in Riyadh only; generalizability to non-tertiary settings (primary care, regional hospitals) and other Saudi regions (Eastern Province, Western Region) is uncertain.

Third, reliance on self-reported communication quality and adherence introduces potential recall and social desirability biases; future studies should incorporate objective measures (e.g., pharmacy claims data, electronic pill counters, prescription refill records) to validate findings and reduce measurement error.

Fourth, the communication quality items were investigator-developed and, while pilot-tested, have not undergone formal validation; the modest internal consistency (Cronbach’s α = 0.72) suggests the items capture related but distinct constructs. Content validity indices (CVI ≥ 0.80) were established through expert panel review, but further validation studies are warranted.

Fifth, we could not compare responders and non-responders on clinical characteristics; thus, selection bias cannot be fully assessed. Sixth, the single-item medication knowledge assessment has limited granularity; more comprehensive assessments examining understanding of medication purpose, dosing, duration, and side effects would be more informative.

Seventh, limiting participation to Arabic or English speakers may have excluded non-Arabic-speaking migrant workers and expatriates in Saudi Arabia; future research should include interpreter services to reduce selection bias and enhance inclusivity.

Finally, the multivariable model explained modest variance (Nagelkerke R^2^ = 0.14), indicating that while communication quality and demographic factors are independent contributors to adherence, substantial additional variance is attributable to unmeasured factors (e.g., medication side effects, health beliefs, provider consistency) that substantially influence adherence behavior.

### 4.3. Implications for Practice and Research

These findings suggest that structured discharge education protocols with explicit attention to side-effect counselling, verification of patient understanding (e.g., teach-back methods), and assessment of home support may warrant evaluation as strategies to improve communication quality. Nurse led interventions have demonstrated effectiveness in improving medication adherence across multiple cardiovascular populations, including those with acute coronary syndrome, stable coronary artery disease, and heart failure [16].

Future research should employ longitudinal designs to clarify temporal relationships between education quality and antiplatelet medication adherence trajectories over 12 months post-intervention and beyond. Intervention studies testing structured education protocols, ideally randomized controlled trials, are needed to establish whether addressing these deficits improves clinical outcomes. Qualitative research exploring patient and provider perspectives on communication barriers could inform the design of culturally tailored interventions.

## 5. Conclusions

This cross-sectional study identifies substantial deficits in patient-reported medication education quality among Saudi post-PCI patients, with particular gaps in side-effect communication and medication understanding. Never receiving side-effect information was independently associated with low adherence after adjustment for sociodemographic and clinical factors.

These findings highlight potentially modifiable targets for nursing intervention. Structured, nurse led educational protocols addressing medication purpose, side effects, and administration could be evaluated as strategies to enhance patient understanding and support antiplatelet adherence outcomes in this high-risk population.

## Figures and Tables

**Table 1:** Sociodemographic and Clinical Characteristics of Participants (*n* = 236).

Characteristic	*n* (%) or Mean ± SD
Age (years)	58.4 ± 11.2
Sex	
Male	174 (73.7)
Female	62 (26.3)
Marital status	
Married	191 (80.9)
Single	18 (7.6)
Divorced	12 (5.1)
Widowed	15 (6.4)
Educational attainment	
No formal schooling	78 (33.1)
General education	107 (45.3)
Higher education	51 (21.6)
Employment status	
Employed	89 (37.7)
Unemployed	62 (26.3)
Retired	85 (36.0)
Monthly household income (SAR)	
<5000	68 (28.8)
5000–10,000	102 (43.2)
>10,000	66 (28.0)
Time since PCI	
≤3 months	72 (30.5)
4–6 months	89 (37.7)
7–12 months	75 (31.8)
PCI indication	
Elective	94 (39.8)
Acute coronary syndrome (ACS)/Emergency	142 (60.2)
Current antiplatelet regimen	
Dual antiplatelet therapy (DAPT: aspirin + P2Y12 inhibitor)	198 (83.9)
Single antiplatelet therapy	38 (16.1)
Pre-existing heart disease	164 (69.5)
Comorbidities	
Hypertension	121 (51.3)
Diabetes mellitus	106 (45.0)
Hyperlipidaemia	89 (37.7)

Abbreviations: ACS, acute coronary syndrome; DAPT, dual antiplatelet therapy; PCI, percutaneous coronary intervention; SAR, Saudi Riyal; SD, standard deviation.

**Table 2:** Quality of Medication-Related Communication (*n* = 236).

Communication Domain	Never *n* (%)	Sometimes *n* (%)	Usually *n* (%)	Always *n* (%)
Clear explanation during hospital stay	16 (6.8)	69 (29.2)	56 (23.7)	95 (40.3)
Informed about purpose of new medication	26 (11.0)	61 (25.8)	56 (23.7)	93 (39.4)
Informed about side effects	62 (26.3)	66 (28.0)	46 (19.5)	62 (26.3)
Understood medication instructions	33 (14.0)	94 (39.8)	45 (19.1)	64 (27.1)
Someone at home helps with medication	71 (30.1)	24 (10.2)	27 (11.4)	114 (48.3)
Sees the same cardiac specialist regularly	44 (18.6)	54 (22.9)	46 (19.5)	92 (39.0)

Note: All items sum to *n* = 236. Percentages may not total 100% due to rounding.

**Table 3:** Antiplatelet Medication Adherence (MMAS-8 Categories, *n* = 233).

Adherence Level	*n*	%
High (score = 8)	23	9.9
Medium (score 6 to <8)	82	35.2
Low (score < 6)	128	54.9
Total	233	100.0

Note: Three participants excluded due to ≥ 2 missing MMAS-8 items.

**Table 4:** Bivariate Associations Between Communication Quality and Low Adherence (*n* = 233).

Communication Indicator	Low Adherence *n* (%)	Medium/High Adherence *n* (%)	χ^2^	*p*	Cramér’s V
Informed about side effects			8.42	0.004	0.19
Never	44 (71.0)	18 (29.0)			
Sometimes/Usually/Always	84 (49.1)	87 (50.9)			
Understood medication instructions			6.18	0.013	0.16
Never	24 (75.0)	8 (25.0)			
Sometimes/Usually/Always	104 (51.7)	97 (48.3)			
Home support available			4.92	0.027	0.15
Never	47 (67.1)	23 (32.9)			
Sometimes/Usually/Always	81 (49.7)	82 (50.3)			
Sees same specialist			3.21	0.073	0.12
Never/Sometimes	59 (60.8)	38 (39.2)			
Usually/Always	69 (50.7)	67 (49.3)			

**Table 5:** Multivariable Logistic Regression: Factors Associated with Low Adherence (*n* = 233).

Variable	Adjusted OR	95% CI	*p*-Value
Never informed about side effects (vs. sometimes/usually/always)	2.14	1.18–3.89	0.012
Never understood instructions (vs. sometimes/usually/always)	1.89	0.91–3.93	0.087
No home support (vs. any support)	1.72	1.01–2.94	0.046
Age (per 10-year increase)	0.92	0.78–1.09	0.34
Female sex (vs. male)	1.18	0.64–2.17	0.59
No formal schooling (vs. higher education)	1.56	0.79–3.08	0.20
General education (vs. higher education)	1.21	0.65–2.26	0.55
Time since PCI 7–12 months (vs. ≤3 months)	1.44	0.81–2.56	0.21
Diabetes mellitus	1.38	0.84–2.27	0.21
Hypertension	1.12	0.68–1.85	0.66

Model fit: Hosmer–Lemeshow χ^2^ = 6.84, *p* = 0.55 (adequate fit); Nagelkerke R^2^ = 0.14. Abbreviations: OR = odds ratio; CI = confidence interval; PCI = percutaneous coronary intervention.

## Data Availability

The datasets generated and analysed during this study are available from the corresponding author on reasonable request. The data are not publicly available due to ethical and privacy restrictions related to participant confidentiality.

## Supplementary Materials

The following supporting information can be downloaded at: https://www.mdpi.com/article/10.3390/healthcare14070891/s1. Table S1: strobe checklist for cross-sectional studies; Table S2: complete questionnaire (English & Arabic); Table S3: medication adherence (MMAS-8); Table S4: sensitivity analyses results.
