# Supplementary material for: Patient Education Deficits and Medication Knowledge Gaps Among Post-Percutaneous Coronary Intervention Patients: A Cross-Sectional Study of Communication Quality and Adherence in Saudi Cardiac Care
Source: Healthcare (Basel). 2026 Mar 31;14(7):891. doi: 10.3390/healthcare14070891 (PMC13073169; doi:10.3390/healthcare14070891)
Supplement: Supplementary file 1 [file healthcare-14-00891-s001.zip › healthcare-4168693-supplementary/Supplementary Table S1.pdf]

SUPPLEMENTARY MATERIALS

SUPPLEMENTARY TABLE S1: STROBE CHECKLIST FOR CROSS-SECTIONAL STUDIES

Strengthening the Reporting of Observational Studies in Epidemiology (STROBE) Statement

Checklist of items that should be included in reports of cross-sectional studies

Item No.	STROBE Item	Recommendation	Reported	Location in Manuscript
**TITLE AND ABSTRACT**				
1	Title and Abstract (a) Indicate the study's design with a commonly used term in the title or abstract	(a) Indicate the study's design	✓	Title: "A Cross-Sectional Study"; Abstract line 1
1	(b) Provide in the abstract an informative and balanced summary of what was done and what was found	(b) Balanced summary	✓	Abstract (all sections)
**INTRODUCTION**				
2	Background/rationale	Explain the scientific background and rationale for the investigation	✓	Introduction, paragraphs 1–5
3	Objectives	State specific objectives, including any prespecified hypotheses	✓	Introduction, final paragraph
**METHODS**				
4	Study design	Present key elements of study design	✓	Section 2.1 "Study Design"

		early in the paper		
5	Setting	Describe the setting, locations, and relevant dates, including periods of recruitment, exposure, follow-up, and data collection	✓	Section 2.2 "Setting and Sample" (January–June 2023; two hospitals described)
6	Participants	(a) Give the eligibility criteria, and the sources and methods of selection of participants	✓	Section 2.2 "Setting and Sample" (eligibility criteria, consecutive sampling)
7	Variables	Clearly define all outcomes, exposures, predictors, potential confounders, and effect modifiers. Give diagnostic criteria, if applicable	✓	Section 2.3 "Data Collection Instruments" (all variables defined); Section 2.5 (covariates for regression)
8	Data sources/measurement	For each variable of interest, give sources of data and details of methods of assessment (measurement). Describe comparability of assessment methods if	✓	Section 2.3 (questionnaire details, MMAS-8, translation process); Supplementary Table S1 (full instrument)

		there is more than one group		
9	Bias	Describe any efforts to address potential sources of bias	✓	Section 2.4 (independent research assistants, private administration); Section 4.2 "Limitations" (bias discussion)
10	Study size	Explain how the study size was arrived at	✓	Section 2.2 "Sample size justification"
11	Quantitative variables	Explain how quantitative variables were handled in the analyses. If applicable, describe which groupings were chosen and why	✓	Section 2.5 "Data Analysis"; Supplementary Table S2 (codebook with derived variables)
12	Statistical methods	(a) Describe all statistical methods, including those used to control for confounding	✓	Section 2.5 "Data Analysis" (descriptive, bivariate, multivariable logistic regression)
12	(b) Describe any methods used to examine subgroups and interactions	✓	Section 2.5 (sensitivity analyses by time since PCI and PCI indication)	
12	(c) Explain how missing data were addressed	✓	Section 2.5 ("no imputation"; MMAS exclusion rule); Supplementar	

			y Table S2 (missing data summary)	
12	(d) If applicable, describe analytical methods taking account of sampling strategy	N/A	Consecutive sampling; no complex survey weights	
12	(e) Describe any sensitivity analyses	✓	Section 2.5; Section 3.10 "Sensitivity Analyses"	
**RESULTS**				
13	Participants	(a) Report numbers of individuals at each stage of study	✓	Section 3.1 "Participant Flow"; Figure 1 (flow diagram)
13	(b) Give reasons for non-participation at each stage	✓	Section 3.1; Figure 1 (reasons for decline)	
13	(c) Consider use of a flow diagram	✓	Figure 1	
14	Descriptive data	(a) Give characteristics of study participants	✓	Table 1 (sociodemograph ic and clinical characteristics)
14	(b) Indicate number of participants with missing data for each variable of interest	✓	Section 3.1 (MMAS missing n=3); Supplementar y Table S2 (missing data summary)	
15	Outcome data	Report numbers of outcome events or summary measures	✓	Table 3 (adherence categories with n and %); Section 3.7
16	Main results	(a) Give unadjusted estimates and,	✓	Table 4 (bivariate with χ^2, p, Cramér's V);

		if applicable, confounder-adjusted estimates and their precision (e.g., 95% CI)		Table 5 (adjusted OR with 95% CI)
16	(b) Report category boundaries when continuous variables were categorized	✓	Codebook (Supplementary Table S2); Table 1 notes	
16	(c) If relevant, consider translating estimates of relative risk into absolute risk	N/A	Cross-sectional; prevalence reported	
17	Other analyses	Report other analyses done—e.g., analyses of subgroups and interactions, and sensitivity analyses	✓	Section 3.10 "Sensitivity Analyses" (stratified by time since PCI and PCI indication)
**DISCUSSION**				
18	Key results	Summarize key results with reference to study objectives	✓	Discussion, paragraph 1
19	Limitations	Discuss limitations of the study, taking into account sources of potential bias or imprecision. Discuss both direction and magnitude of any potential bias	✓	Section 4.2 "Strengths and Limitations" (6 specific limitations with direction of bias)

20	Interpretation	Give a cautious overall interpretation of results considering objectives, limitations, multiplicity of analyses, results from similar studies, and other relevant evidence	✓	Section 4.1 "Key Findings in Context"; Section 4.3 "Implications"
21	Generalisability	Discuss the generalisability (external validity) of the study results	✓	Section 4.2 Limitations ("generalizability to other regions...uncertain")
**OTHER INFORMATION**				
22	Funding	Give the source of funding and the role of the funders for the present study and, if applicable, for the original study on which the present article is based	✓	Funding statement (IMSIU grant; funders had no role)
