# Supplementary material for: Patient Education Deficits and Medication Knowledge Gaps Among Post-Percutaneous Coronary Intervention Patients: A Cross-Sectional Study of Communication Quality and Adherence in Saudi Cardiac Care
Source: Healthcare (Basel). 2026 Mar 31;14(7):891. doi: 10.3390/healthcare14070891 (PMC13073169; doi:10.3390/healthcare14070891)
Supplement: Supplementary file 1 [file healthcare-14-00891-s001.zip › healthcare-4168693-supplementary/Supplementary Table S2.pdf]

Supplementary Table S2

COMPLETE QUESTIONNAIRE (ENGLISH & ARABIC

SECTION A: SOCIODEMOGRAPHIC INFORMATION

Item	English	Arabic
A1	What is your age (in years)? ______	كم عمرك (بالسنوات)؟ ______
A2	What is your sex? ☐ Male ☐ Female	ما هو جنسك؟ ☐ ذكر ☐ أنثى
A3	What is your current marital status? ☐ Single ☐ Married ☐ Divorced ☐ Widowed	ما هو وضعك الحالي؟ ☐ أعزب/عزباء ☐ متزوج/متزوجة ☐ مطلق/مطلقة ☐ أرمل/أرملة
A4	What is your highest level of education? ☐ No formal schooling ☐ Primary school ☐ Intermediate school ☐ Secondary school ☐ Diploma ☐ Bachelor's degree ☐ Postgraduate degree	ما أعلى مستوى تعليمي لك؟ ☐ لم أتلقَ تعليماً رسمياً ☐ المرحلة الابتدائية ☐ المرحلة المتوسطة ☐ المرحلة الثانوية ☐ دبلوم ☐ درجة البكالوريوس ☐ درجة الدراسات العليا
A5	What is your current employment status? ☐ Employed (full-time) ☐ Employed (part-time) ☐ Self-employed ☐ Unemployed ☐ Retired ☐ Unable to work due to health	ما هو وضعك الوظيفي الحالي؟ ☐ موظف (دوام كامل) ☐ موظف (دوام جزئي) ☐ عامل حر ☐ عاطل عن العمل ☐ متقاعد ☐ غير قادر على العمل لأسباب صحية
A6	What is your approximate monthly household income? ☐ Less than 5,000 SAR ☐ 5,000–10,000 SAR ☐ 10,001–15,000 SAR ☐ More than 15,000 SAR ☐ Prefer not to answer	ما هو الدخل الشهري التقريبي لأسرتك؟ ☐ أقل من 5,000 ريال ☐ 5,000–10,000 ريال ☐ 10,001–15,000 ريال ☐ أكثر من 15,000 ريال ☐ أفضل عدم الإجابة

SECTION B: CLINICAL INFORMATION

Item	English	Arabic
B1	How long ago did you have your most recent heart stent procedure (PCI)? ☐ Less than 3 months ago ☐ 3 to 6 months ago ☐ 7 to 12 months ago	كم مضى على إجراء أحدث عملية لتركيب دعامة بالقلب (PCI) ؟ ☐ أقل من 3 أشهر ☐ من 3 إلى 6 أشهر ☐ من 7 إلى 12 شهراً
B2	Was your heart stent procedure: ☐ Planned/Elective (scheduled in advance) ☐ Emergency (due to heart attack or urgent symptoms) ☐ I don't know	هل كانت عملية تركيب الدعامة: ☐ مخطط لها مسبقاً ☐ طارئة (بسبب جلطة قلبية أو أعراض طارئة) ☐ لا أعرف
B3	Did you have heart disease before this procedure? ☐ Yes ☐ No ☐ I don't know	هل كان لديك مرض بالقلب قبل هذه العملية؟ ☐ نعم ☐ لا ☐ لا أعرف
B4	Do you have any of the following conditions? (Check all that apply) ☐ High blood pressure (Hypertension) ☐ Diabetes ☐ High cholesterol	هل لديك أي من الحالات التالية؟ (اختر كل ما ينطبق) ☐ ارتفاع ضغط الدم ☐ السكري ☐ ارتفاع الكوليسترول ☐ أمراض الكلى ☐ لا شيء من السابق

	(Hyperlipidemia) ☐ Kidney disease ☐ None of the above	
B5	Which blood-thinning (antiplatelet) medications are you currently taking? (Check all that apply) ☐ Aspirin (Aspocid, Juspirin) ☐ Clopidogrel (Plavix) ☐ Ticagrelor (Brilinta) ☐ Prasugrel (Effient) ☐ I don't know the names	ما هي الأدوية المميعة للدم (مانعات التجلط) التي تتناولها الآن؟ (اختر كل ما ينطبق) ☐ أسبرين (أسبوسيد، جسبرين) ☐ كلوبيدوجريل (بلافكس) ☐ تيكاجريلور (بريلينتا) ☐ براسوجريل (إيفيينت) ☐ لا أعرف أسماء الأدوية

SECTION C: MEDICATION-RELATED COMMUNICATION QUALITY

Instructions: Thinking about your care since your heart procedure, please indicate how often the following occurred.

Item	English	Arabic	Response Options
C1	How often did healthcare providers give you clear explanations about your medications during your hospital stay?	كم مرة أعطاك مقدمو الرعاية الصحية شرحاً واضحاً عن أدويتك أثناء إقامتك في المستشفى؟	☐ Never ☐ Sometimes ☐ Usually ☐ Always
C2	How often did healthcare providers tell you the purpose of any new medications?	كم مرة أخبرك مقدمو الرعاية الصحية بالغرض من أي أدوية جديدة؟	☐ Never ☐ Sometimes ☐ Usually ☐ Always
C3	How often did healthcare providers explain possible side effects of your medications?	كم مرة شرح لك مقدمو الرعاية الصحية الآثار الجانبية المحتملة لأدويتك؟	☐ Never ☐ Sometimes ☐ Usually ☐ Always
C4	How often did healthcare providers make sure you understood your medication instructions before leaving the hospital?	كم مرة تأكد مقدمو الرعاية الصحية من أنك فهمت تعليمات أدويتك قبل مغادرة المستشفى؟	☐ Never ☐ Sometimes ☐ Usually ☐ Always
C5	How often did healthcare providers ask whether you have someone at home to help you take your medications?	كم مرة سأل مقدمو الرعاية الصحية عما إذا كان لديك شخص في المنزل يساعدك على تناول الأدوية؟	☐ Never ☐ Sometimes ☐ Usually ☐ Always
C6	How often do you see the same cardiac specialist at your follow-up visits?	كم مرة ترى نفس اختصاصي القلب في زيارات المتابعة الخاصة بك؟	☐ Never ☐ Sometimes ☐ Usually ☐ Always

SECTION D: MEDICATION KNOWLEDGE

Item	English	Arabic
D1	What is the name of the blood-thinning (antiplatelet) medication you are currently	ما هو اسم الدواء المميع للدم (مانع التجلط) الذي تتناوله حالياً لحماية دعامة القلب؟ يرجى كتابة

	taking to protect your heart stent? Please write the name(s): _________________ ☐ I don't know	الاسم (الأسماء): _________________ ☐ لا أعرف
D2	Do you know how long you need to continue taking your blood-thinning medications? ☐ Yes, I know: _____ months/years ☐ For the rest of my life ☐ No, I don't know	هل تعرف كم مدة استمرارك على تناول أدوية تميع الدم؟ ☐ نعم، أعرف: _____ شهور/سنوات ☐ لبقية حياتي ☐ لا، لا أعرف

SECTION E: MEDICATION ADHERENCE (MMAS-8)

Note: The Morisky Medication Adherence Scale-8 (MMAS-8) is a copyrighted instrument. Use requires permission from MMAS Research LLC.

Item No.	Domain Description	Response Format
E1	Forgetting to take medication	Yes/No
E2	Missing doses in past two weeks	Yes/No
E3	Stopping medication when feeling worse	Yes/No
E4	Stopping medication when feeling better	Yes/No
E5	Forgetting to take medication yesterday	Yes/No
E6	Stopping medication when symptoms are under control	Yes/No
E7	Feeling hassled about sticking to treatment plan	Yes/No
E8	Difficulty remembering to take all medications	5-point Likert scale

Scoring:

- Items E1–E7 scored as No=1, Yes=0
- Item E8 scored on 5-point scale (Never/Rarely=1; Once in a while=0.75; Sometimes=0.5; Usually=0.25; All the time=0)
- Total score range: 0–8
- **High adherence:** Score = 8
- **Medium adherence:** Score 6 to <8
- **Low adherence:** Score <6
