# Supplementary material for: Patient Education Deficits and Medication Knowledge Gaps Among Post-Percutaneous Coronary Intervention Patients: A Cross-Sectional Study of Communication Quality and Adherence in Saudi Cardiac Care
Source: Healthcare (Basel). 2026 Mar 31;14(7):891. doi: 10.3390/healthcare14070891 (PMC13073169; doi:10.3390/healthcare14070891)
Supplement: Supplementary file 1 [file healthcare-14-00891-s001.zip › healthcare-4168693-supplementary/Supplementary Table S3.pdf]

Supplementary Table S3

MEDICATION ADHERENCE (MMAS-8)

Note: The Morisky Medication Adherence Scale-8 (MMAS-8) is a copyrighted instrument. Use requires permission from MMAS Research LLC.

Item No.	Domain Description	Response Format
E1	Forgetting to take medication	Yes/No
E2	Missing doses in past two weeks	Yes/No
E3	Stopping medication when feeling worse	Yes/No
E4	Stopping medication when feeling better	Yes/No
E5	Forgetting to take medication yesterday	Yes/No
E6	Stopping medication when symptoms are under control	Yes/No
E7	Feeling hassled about sticking to treatment plan	Yes/No
E8	Difficulty remembering to take all medications	5-point Likert scale

Scoring:

- Items E1–E7 scored as No=1, Yes=0
- Item E8 scored on 5-point scale (Never/Rarely=1; Once in a while=0.75; Sometimes=0.5; Usually=0.25; All the time=0)
- Total score range: 0–8
- **High adherence:** Score = 8
- **Medium adherence:** Score 6 to <8
- **Low adherence:** Score <6
