# Supplementary material for: Patient Education Deficits and Medication Knowledge Gaps Among Post-Percutaneous Coronary Intervention Patients: A Cross-Sectional Study of Communication Quality and Adherence in Saudi Cardiac Care
Source: Healthcare (Basel). 2026 Mar 31;14(7):891. doi: 10.3390/healthcare14070891 (PMC13073169; doi:10.3390/healthcare14070891)
Supplement: Supplementary file 1 [file healthcare-14-00891-s001.zip › healthcare-4168693-supplementary/Supplementary Table S4.pdf]

SUPPLEMENTARY TABLE S4: SENSITIVITY ANALYSES RESULTS

A. STRATIFIED ANALYSES BY TIME SINCE PCI

≤3 MONTHS POST-PCI (n=72)

Variable	Adjusted OR	95% CI	p-value	N with Low Adherence (%)	Model Fit
Never informed about side effects (vs. sometimes/usually/always)	2.31	0.89–6.01	0.087	31/44 (70.5%)	Hosmer–Lemeshow χ^2=3.21, p=0.67 (adequate); Nagelkerke R^2=0.18
Never understood instructions (vs. sometimes/usually/always)	1.78	0.63–5.02	0.279	12/18 (66.7%)	
No home support (vs. any support)	1.58	0.67–3.72	0.299	25/38 (65.8%)	
Age (per 10-year increase)	0.89	0.71–1.12	0.320	—	
Female sex (vs. male)	1.24	0.53–2.91	0.620	—	

Clinical Interpretation: Among patients ≤3 months post-PCI, the point estimate for side-effect education association (aOR 2.31) is slightly elevated compared to overall sample (aOR 2.14), but the 95% confidence interval widens and crosses unity, reflecting smaller subgroup sample size (n=72). Numerically, 70.5% of patients never receiving side-effect information demonstrated low adherence. This early post-PCI period represents an important intervention opportunity before adherence patterns become entrenched.

4–6 MONTHS POST-PCI (n=89)

Variable	Adjusted OR	95% CI	p-value	N with Low Adherence (%)	Model Fit
Never informed about side effects (vs. sometimes/usually/always)	2.07	0.87–4.92	0.102	38/61 (62.3%)	Hosmer–Lemeshow χ^2=2.89, p=0.72 (adequate);

					Nagelkerke R^2=0.16
Never understood instructions (vs. sometimes/usually/always)	1.94	0.71–5.30	0.194	14/21 (66.7%)	
No home support (vs. any support)	1.84	0.78–4.35	0.167	29/46 (63.0%)	
Age (per 10-year increase)	0.91	0.75–1.10	0.330	—	
Female sex (vs. male)	1.21	0.58–2.53	0.610	—	

Clinical Interpretation: At 4–6 months post-PCI (the "consolidation phase"), the association remains consistent with the overall sample (aOR 2.07 vs. main 2.14), suggesting a trend toward statistical significance (p=0.102). This intermediate timeframe shows 62.3% low adherence among those not receiving side-effect education. The pattern suggests education deficits have cumulative effects on adherence as time since intervention increases.

7–12 MONTHS POST-PCI (n=75)

Variable	Adjusted OR	95% CI	p-value	N with Low Adherence (%)	Model Fit
Never informed about side effects (vs. sometimes/usually/always)	2.39	1.03–5.57	0.043*	26/41 (63.4%)	Hosmer–Lemeshow χ^2=3.15, p=0.69 (adequate); Nagelkerke R^2=0.17
Never understood instructions (vs. sometimes/usually/always)	2.08	0.79–5.49	0.141	15/22 (68.2%)	
No home support (vs. any support)	1.65	0.71–3.83	0.252	20/38 (52.6%)	
Age (per 10-year increase)	0.94	0.77–1.15	0.540	—	
Female sex (vs. male)	1.12	0.51–2.47	0.780	—	

Clinical Interpretation: Among patients 7–12 months post-PCI, the association between never receiving side-effect information and low adherence reaches statistical significance (aOR 2.39, p=0.043*), representing the strongest effect observed across all time strata. The narrowing

confidence interval (1.03–5.57) and lower p-value indicate a more stable, clinically meaningful effect size at longer follow-up. By 7–12 months, educational gaps show clear association with adherence failure, suggesting that early education intervention is critical before non-adherence patterns become established.

SUMMARY—TIME SINCE PCI STRATIFICATION:

Time Stratum	aOR (95% CI)	p-value	Clinical Significance
≤3 months (n=72)	2.31 (0.89–6.01)	0.087	Trend; wider CI
4–6 months (n=89)	2.07 (0.87–4.92)	0.102	Trend; consistent
7–12 months (n=75)	2.39 (1.03–5.57)	0.043*	Statistically significant

Key Finding: Effect size strengthens over time, with point estimates ranging 2.07–2.39. Statistical significance achieved only at 7–12 months (aOR 2.39, p=0.043*). Interpretation: Education deficits are consistently associated with adherence across all timeframes, but the relationship strengthens and becomes clearly statistically significant at longer follow-up periods, suggesting cumulative impact.

B. STRATIFIED ANALYSES BY PCI INDICATION

ELECTIVE PCI (n=94)

Variable	Adjusted OR	95% CI	p-value	N with Low Adherence (%)	Model Fit
Never informed about side effects (vs. sometimes/usually/always)	1.89	0.72–4.96	0.197	22/37 (59.5%)	Hosmer–Lemeshow χ^2=4.32, p=0.50 (adequate); Nagelkerke R^2=0.12
Never understood instructions (vs. sometimes/usually/always)	1.64	0.56–4.79	0.364	8/13 (61.5%)	
No home support (vs. any support)	1.51	0.61–3.73	0.378	18/35 (51.4%)	
Age (per 10-year increase)	0.95	0.77–1.18	0.630	—	
Female sex (vs. male)	1.31	0.55–3.12	0.540	—	

Diabetes mellitus	1.22	0.62–2.40	0.570	—	

Clinical Interpretation: Among patients undergoing elective (planned) PCI procedures (n=94), the association between side-effect education deficits and low adherence is weaker (aOR 1.89, p=0.197, non-significant) compared to the overall sample (aOR 2.14, p=0.012, significant). Several factors may explain this:

- Elective PCI patients typically have more time before the procedure, allowing more comprehensive education
- Elective patients may have higher baseline health literacy and engagement
- Elective procedures may select for more motivated/adherent patients
- Lower adherence prevalence in elective group (59.5% vs. 75% in emergency) suggests selection effects

EMERGENCY/ACS PCI (n=142)

Variable	Adjusted OR	95% CI	p-value	N with Low Adherence (%)	Model Fit
Never informed about side effects (vs. sometimes/usually/always)	2.48	1.18–5.19	0.017*	36/48 (75.0%)	Hosmer–Lemeshow χ^2=5.21, p=0.51 (adequate); Nagelkerke R^2=0.15
Never understood instructions (vs. sometimes/usually/always)	2.14	0.87–5.26	0.096	17/25 (68.0%)	
No home support (vs. any support)	1.89	0.99–3.60	0.053	29/60 (48.3%)	
Age (per 10-year increase)	0.90	0.73–1.11	0.320	—	
Female sex (vs. male)	1.15	0.57–2.34	0.690	—	
Diabetes mellitus	1.51	0.84–2.72	0.170	—	

Clinical Interpretation: Among patients undergoing emergency/ACS PCI presentations (n=142, 60.2% of sample), the association between never receiving side-effect information and low

adherence is significantly stronger (aOR 2.48, p=0.017*) compared to elective procedures (aOR 1.89, p=0.197). Key observations:

- Statistically significant: The association reaches significance in emergency subgroup but not elective, indicating differential impact
- High prevalence: 75% of emergency PCI patients never receiving side-effect education demonstrated low adherence, the highest rate observed
- Vulnerable population: Emergency/ACS patients have acute onset, limited pre-procedure education time, and potentially higher stress, making them particularly vulnerable to education gaps
- Home support significance: Trend toward significance for home support (p=0.053) suggests emergency PCI patients particularly benefit from caregiver involvement
- Clinical implications: Emergency presentations preclude comprehensive pre-procedure education, placing greater emphasis on post-intervention discharge counselling

COMPARISON ACROSS PCI INDICATION STRATA:

PCI Indication	Never Informed Side Effects: aOR (95% CI)	p-value	Low Adherence %	Clinical Significance
Elective (n=94)	1.89 (0.72–4.96)	0.197	59.5%	Non-significant trend
Emergency/ACS (n=142)	2.48 (1.18–5.19)	0.017*	75.0%	Statistically significant
Difference	+0.59 (aOR)	0.017 vs 0.197	+15.5%	Stronger in emergency

Key Finding: The association between side-effect education deficits and low adherence is 31% stronger in emergency presentations (aOR 2.48 vs. 1.89) and achieves statistical significance only in the ACS/emergency subgroup. Clinical Implication: Education deficits disproportionately impact patients with acute presentations, suggesting this vulnerable population requires prioritized intervention.

C. COMBINED STRATIFICATION: TIME × PCI INDICATION

Matrix of aOR estimates (side-effect education deficit):

Time Since PCI	Elective (aOR, p)	Emergency/ACS (aOR, p)	Combined Effect
≤3 months	1.87 (0.214)	2.64 (0.063)	2.25*
4–6 months	1.92 (0.186)	2.31 (0.047*)	2.11*

7–12 months	2.15 (0.112)	2.58 (0.019*)	2.36*

Interpretation: The strongest associations consistently occur in emergency/ACS presentations, regardless of time stratum. Effect strengthens in both groups over time, with 7–12 month emergency subgroup showing aOR 2.58 (p=0.019*).

D. SENSITIVITY ANALYSIS: ORDINAL ADHERENCE OUTCOMES

Alternative analytical approach: Three-level adherence categorization (low, medium, high) examined using ordinal logistic regression to test robustness of dichotomized findings.

Variable	Proportional Odds Ratio	95% CI	p-value	Brant Test (p)	Assumption
Never informed about side effects	1.98	1.12–3.50	0.019*	0.34	Not violated
No home support	1.64	0.98–2.75	0.058	0.71	Not violated
Never understood instructions	1.81	0.89–3.68	0.105	0.52	Not violated
Overall Model	Brant test χ^2=4.23, p=0.645	—	—	—	All assumptions satisfied

Interpretation: Proportional odds regression (treating adherence as ordinal: low < medium < high) yields similar results to binary logistic regression. Brant test confirms proportional odds assumption is satisfied (p values >0.05 across all variables), validating the dichotomized primary approach. Conclusion: Results are robust to different adherence categorization schemes.

E. SENSITIVITY ANALYSIS: MISSING DATA IMPACT (MULTIPLE IMPUTATION)

Approach: Multiple imputation (5 imputations; MCAR assumption) to assess bias from 3 cases with ≥2 missing MMAS-8 items.

Analysis Approach	Never Informed Side Effects: aOR (95% CI)	p-value	N	Change from CC
Complete Case (Primary)	2.14 (1.18–3.89)	0.012	233	—
Multiple Imputation (5 imp.)	2.11 (1.15–3.87)	0.015	236	−0.03 (−1.4%)

Difference	—	—	—	Negligible

Model Fit Comparison:

- Complete Case: Nagelkerke $R^2 = 0.14$
- Multiple Imputation: Nagelkerke $R^2 = 0.14$ (identical)

Interpretation: Multiple imputation of the 3 missing cases yields aOR 2.11 (95% CI 1.15–3.87, p=0.015), virtually identical to the complete-case analysis (aOR 2.14, difference = −1.4%). Conclusion: The minimal missing data (<2% of sample) does not bias results. Complete-case analysis is appropriate and recommended.

F. SENSITIVITY ANALYSIS: ALTERNATIVE DICHOTOMIZATION SCHEMES FOR ADHERENCE

Rationale: Explore robustness of primary dichotomization (low <6 vs. ≥6) using alternative cut-points based on different clinical interpretations of MMAS-8 scores.

Adherence Cut-Point	Outcome Definition	Never Informed Side Effects: aOR (95% CI)	p-value	N	Clinical Rationale
≤5 vs. ≥6 (PRIMARY)	Low (<6) vs. Medium/High (≥6)	2.14 (1.18–3.89)	0.012*	233	Standard MMAS-8; distinguishes non-adherent from adequate
<7 vs. ≥7	Low/Medium (<7) vs. High (≥7)	1.92 (0.95–3.88)	0.072	233	More stringent; includes medium as suboptimal
<8 vs. = 8	Low/Medium (<8) vs. High (=8)	1.78 (0.81–3.91)	0.148	233	Strictest; only perfect adherence as "high"
0–2 vs. >2	Very Low vs. Higher	2.41 (1.29–4.50)	0.006*	233	Most lenient; compares most vs. least adherent

Interpretation:

- Primary dichotomization (low <6 vs. ≥6) achieves statistical significance (p=0.012)
- Effect weakens with stricter definitions (aOR 1.78, p=0.148) as fewer patients categorized as "high"
- Effect strengthens with most lenient definition (aOR 2.41, p=0.006*)

- Conclusion: Primary dichotomization is optimal; it aligns with established MMAS-8 clinical cut-points and provides clearest distinction of clinically meaningful adherence levels

G. SENSITIVITY ANALYSIS: DEMOGRAPHIC SUBGROUP ANALYSES

By Age Groups

Age Group	N	Never Informed Side Effects: aOR (95% CI)	p-value	Low Adherence %	Interpretation
<50 years	62	1.87 (0.71–4.91)	0.210	48/62 (77.4%)	Weak association; high overall adherence problems
50–65 years	108	2.31 (1.02–5.22)	0.045*	55/108 (50.9%)	Strongest association; significant
>65 years	63	2.18 (0.84–5.67)	0.108	25/63 (39.7%)	Trend; better adherence overall

Key Finding: The association is strongest and statistically significant in the 50–65 year age stratum (aOR 2.31, p=0.045*). Younger patients (<50) show paradoxically high overall adherence problems (77.4%) despite weaker association with education, suggesting multiple adherence barriers in this age group. Older patients (>65) demonstrate improved adherence overall (60.3% adequate), possibly reflecting selection bias or accumulated disease experience.

By Educational Attainment

Education Level	N	Never Informed Side Effects: aOR (95% CI)	p-value	Low Adherence %	Interpretation
No formal schooling	78	2.52 (1.04–6.12)	0.041*	48/78 (61.5%)	Strongest; statistically significant
General education	107	2.18 (1.01–4.70)	0.046*	54/107 (50.5%)	Statistically significant
Higher education	48	1.54 (0.52–4.57)	0.431	18/48 (37.5%)	Weak; much better adherence

Key Finding: The association between side-effect education deficits and low adherence is most pronounced in patients with lower educational attainment. No formal schooling: aOR 2.52 (p=0.041*); General education: aOR 2.18 (p=0.046*); Higher education: aOR 1.54 (non-significant). Clinical Implication: Education gaps disproportionately impact vulnerable

populations with lower baseline health literacy, highlighting the importance of tailored, accessible educational approaches for these groups.

By Presence of Diabetes Mellitus

Diabetes Status	N	Never Informed Side Effects: aOR (95% CI)	p-value	Low Adherence %	Interpretation
Diabetes (Yes)	106	2.39 (1.07–5.34)	0.033*	56/106 (52.8%)	Statistically significant
Diabetes (No)	127	1.95 (0.91–4.17)	0.086	72/127 (56.7%)	Trend; larger CI

Key Finding: Among diabetic patients, the association between side-effect education deficits and low adherence is statistically significant (aOR 2.39, p=0.033*). Diabetic patients typically manage complex polypharmacy and may be more sensitive to educational gaps. Non-diabetic patients show a trend (aOR 1.95, p=0.086), indicating education deficits affect all patients but with heightened impact in those with comorbidity burden.

H. SENSITIVITY ANALYSIS: COMMUNICATION QUALITY SCORE (CONTINUOUS vs. DICHOTOMIZED)

Alternative approach: Investigate whether continuous communication quality index (mean of 6 communication items on 1–4 scale) improves model fit compared to dichotomized individual items.

Model Specification	Predictor Type	Nagelkerke R^2	AIC	Hosmer-Lemeshow p	Advantage
Model 1 (Primary)	Six dichotomized items (Never vs. Any)	0.14	298.4	0.55	Better fit; clinically relevant
Model 2 (Exploratory)	Continuous communication index (mean 1–4)	0.13	301.2	0.48	Continuous scale; less specific
Difference	—	−0.01	+2.8	—	Model 1 superior

Interpretation: The primary analytical approach (dichotomized individual items) provides superior model fit:

- Lower AIC (298.4 vs. 301.2): Better model efficiency
- Higher R^2 (0.14 vs. 0.13): More variance explained
- Clinical relevance: Dichotomization isolates patients receiving zero education ("Never") from all others, capturing the most clinically meaningful distinction
- Specificity: Individual items allow identification of specific communication gaps (side effects, purpose, understanding) rather than global communication quality

Conclusion: Primary analysis using dichotomized individual items is methodologically superior and clinically more informative.

I. SENSITIVITY ANALYSIS: SITE-STRATIFIED ANALYSIS (KING KHALID vs. KING FAHAD)

Hospital	N	Never Informed Side Effects: aOR (95% CI)	p-value	Low Adherence %	Comparison
King Khalid University Hospital	124	2.06 (1.01–4.19)	0.047	66/124 (53.2%)	Significant
King Fahad Medical City	109	2.27 (1.08–4.76)	0.031	62/109 (56.9%)	Significant
Combined (Primary)	233	2.14 (1.18–3.89)	0.012	128/233 (55.0%)	Significant

Interpretation: Both hospitals show consistent associations (King Khalid aOR 2.06, p=0.047; King Fahad aOR 2.27, p=0.031), with point estimates similar to overall sample (aOR 2.14). This consistency across both facilities supports the robustness of findings and indicates that education deficits are systemic within Saudi tertiary cardiac care, not localized to a single institution.

SUMMARY TABLE: ALL SENSITIVITY ANALYSES

Analysis Type	Finding	Robustness	Conclusion
Time since PCI	aOR 2.07–2.39; strongest at 7–12 months (p=0.043*)	✓ Strong	Effect strengthens over time
PCI Indication	Elective aOR 1.89; Emergency aOR 2.48 (p=0.017*)	✓ Strong	Stronger in acute presentations
Ordinal Adherence	POR 1.98 (p=0.019*); assumptions satisfied	✓ Strong	Robust to ordinal categorization
Multiple Imputation	Minimal change (−1.4% from CC); aOR 2.11	✓ Strong	Not biased by missing data

Alternative Dichotomizations	Range aOR 1.78–2.41; primary is optimal	✓ Strong	Primary dichotomization appropriate
Age Stratification	Strongest at 50–65 years (aOR 2.31, p=0.045*)	✓ Moderate	Meaningful effect in middle-aged
Education Stratification	Strongest in no schooling (aOR 2.52, p=0.041*)	✓ Strong	Vulnerable populations at risk
Diabetes Stratification	Significant in diabetics (aOR 2.39, p=0.033*)	✓ Strong	Comorbidity amplifies effect
Continuous vs. Dichotomized	Dichotomized model superior (AIC 298.4 vs. 301.2)	✓ Strong	Primary approach optimal
Site Stratification	Consistent at both hospitals (aOR ~2.1–2.3)	✓ Strong	Systemic, not localized
